# Prevention of biofilm formation by quorum quenching

**DOI:** 10.1007/s00253-020-10349-w

**Published:** 2020-01-11

**Authors:** E. Paluch, J. Rewak-Soroczyńska, I. Jędrusik, E. Mazurkiewicz, K. Jermakow

**Affiliations:** 1 Department of Microbiology, Faculty of Medicine, Wroclaw Medical University, Tytusa Chałubińskiego 4, 50-376 Wrocław, Poland; 2 Institute of Low Temperature and Structure Research, Polish Academy of Science, Okólna 2, 50-422 Wroclaw, Poland; 3 Institute of Genetics and Microbiology, University of Wrocław, Przybyszewskiego 63/77, 51-148 Wrocław, Poland

**Keywords:** Biofilm, Quorum quenching, Quorum sensing, Research methods

## Abstract

Quorum sensing (QS) is a mechanism that enables microbial communication. It is based on the constant secretion of signaling molecules to the environment. The main role of QS is the regulation of vital processes in the cell such as virulence factor production or biofilm formation. Due to still growing bacterial resistance to antibiotics that have been overused, it is necessary to search for alternative antimicrobial therapies. One of them is quorum quenching (QQ) that disrupts microbial communication. QQ-driving molecules can decrease or even completely inhibit the production of virulence factors (including biofilm formation). There are few QQ strategies that comprise the use of the structural analogues of QS receptor autoinductors (AI). They may be found in nature or be designed and synthesized via chemical engineering. Many of the characterized QQ molecules are enzymes with the ability to degrade signaling molecules. They can also impede cellular signaling cascades. There are different techniques used for testing QS/QQ, including chromatography-mass spectroscopy, bioluminescence, chemiluminescence, fluorescence, electrochemistry, and colorimetry. They all enable qualitative and quantitative measurements of QS/QQ molecules. This article gathers the information about the mechanisms of QS and QQ, and their effect on microbial biofilm formation. Basic methods used to study QS/QQ, as well as the medical and biotechnological applications of QQ, are also described. Basis research methods are also described as well as medical and biotechnological application.

## Introduction

In the environment, bacteria rarely occur as a planktonic form that is frequently exposed to many adverse factors. The ability to produce biofilm, i.e., the spatial structure surrounded by the extracellular matrix (EPS), increases protection of microbial cells against harmful factors (Miquel et al. 2016). In the biofilm formation process, stages such as initial adhesion, irreversible adhesion, spatial structure formation, maturation, and final dispersion can be distinguished. Bacterial adhesion to the surfaces as well as cohesion (cell-cell interaction) may occur due to the presence of some bacterial appendages such as flagella or pili or via physical factors like van der Waal’s forces or electrostatic interactions. Biological membranes can be formed on both abiotic and biotic surfaces. The properties of some surfaces facilitate microbial binding. Bacterial adhesion to hydrophobic and non-polar surfaces is characterized by greater binding strength compared with hydrophilic ones because it is suggested that the repulsion force between the surface and the bacteria is reduced (Jamal et al. 2018). Colonization is also affected by surface structure. The size of contact area between bacteria and material is very important in the adhesion process thus rough surfaces (bigger contact area) are more likely colonized than smooth ones (smaller contact area). Shear forces are also reduced on the rough surfaces so adhering cells are protected from detachment (Song et al. 2015). One strategy to inhibit biofilm formation is the prevention of bacterial adhesion to different surfaces. That may be obtained by coating surfaces with antimicrobial agents such as metal-based nanoparticles, surfactants (e.g., quaternary ammonium salts (QAS)), or others. Adhesion reduction was observed for *S. epidermidis* and *C. albicans* cells incubated on pre-treated with dicephalic QAS glass, stainless steel, and silicone surfaces; thus, such compounds may be used to produce resistant to bacterial adhesion medical tools (e.g., catheters) what can lower a risk of nosocomial infections (Paluch et al. 2018; Piecuch et al. 2016). Moreover such compounds are able to decrease the ability to bacterial biofilm production on different metal surfaces, so they may be applied as anti-corrosive and anti-biofilm products (e.g., paints) to protect objects (such as ships, pipes) from degradation (Piecuch et al. 2016; Paluch et al. 2018). A fully developed, mature biofilm is very difficult to eradicate. It is estimated that such microorganism communities are responsible for about 80% of cases of bacterial infections (Jamal et al. 2018). Bacterial biofilms are difficult to control and show high resistance to antibiotics (Koo et al. 2017). For eradication of fully formed biofilm it is necessary to use compounds that are able to penetrate its structure or can disrupt it mechanically. Such activity may be also observed for some surfactants. Sometimes there are not strong enough to eradicate biofilm completely but they lead to cellular death (Rewak-Soroczyńska et al. 2019). The formation of bacterial biofilm by some pathogenic and opportunistic pathogens is under the control of the communication system—quorum sensing (Ding et al. 2011; Li et al. 2018).

The bacterial quorum sensing system is based on the production, release, and detection of extracellular chemical signaling molecules, the so-called autoinductors (Whiteley et al. 2017). These signals accumulate locally in the environment, and then, after reaching the appropriate threshold concentration, interact with the receptor protein leading to coordinated changes in the expression of specific genes (Abisado et al. 2018). Thanks to this, many types of pathogenic bacteria can adapt to different environments regulating the genes responsible for the production of biofilms, virulence factors, antibiotics, or the transfer of genetic material in the process of transformation or conjugation (Reuter et al. 2016). In Gram-negative bacteria, the role of autoinductors is played by N-acylated homoserine lactones (AHLs), synthesized by a *LuxI* type enzyme. These molecules penetrate the bacterial cell membrane, and the number of proliferating cells determines the density of the bacterial population. After reaching the appropriate threshold concentration, the LuxR receptor protein is activated and transcription of target effector genes occurs. An example of the use of the QS system in Gram-negative bacteria is the bacterium *Pseudomonas aeruginosa* in which there are two pairs of *LuxI*/*LuxR* homologs—*LasI*/*LasR* and RhlI/RhlR. In this bacterium, the quorum sensing system controls the formation of biofilm and the expression of many virulence factors such as elastase, protease, alkaline phosphatase, and exotoxin A. Another example is *Vibrio fischeri* where QS system is under the regulation of lux AB genes responsible for luciferase coding and the lux CDE genes encoding enzymes that produce substrates for luciferase, leading to bioluminescence (Nazzaro et al. 2013). Gram-positive bacteria use short oligopeptide signals and two-component systems consisting of membrane-bound sensor kinase receptors and cytoplasmic transcription factors responsible for changing gene expression (Papenfort and Bassler 2016). An example of a Gram-positive bacterium using the quorum sensing system is *Staphylococcus aureus* with an *agr* system that controls the production of virulence factors such as exotoxins or biofilm (LaSarre and Federle 2013).

Resistance of microorganisms to commonly used antibacterial agents is becoming an increasing problem in medicine. Newly developed drugs that were supposed to prevent the emergence of resistance are also beginning to lose their effectiveness against some bacterial strains. For this reason, it is extremely important to search for new antimicrobial therapies that are effective against resistant microorganisms and possess long-term effectiveness. Recent strategies mainly focus on the targeting bacterial cell components that enable the production of virulence factors which is a different approach than the previously applied strategy to inhibit cell growth. This review describes quorum quenching as a relatively new method used to inhibit the production of virulence factors and to prevent biofilm formation by dampening quorum sensing, which interferes with the production of virulence factors involved in quorum sensing.

## Inhibition of QS

Difficulty to remove biofilms and increasing antibiotic resistance necessitates the search for new ways to combat undesirable microorganisms. A promising strategy is to target the QS system. In the environment, there are many compounds that affect the communication between microbes (Rehman and Leiknes 2018). Based on their molecular weight and chemical composition, the compounds fall into one of the groups: macromolecular QQ enzymes and microparticulate QS inhibitors (Tang and Zhang 2014).

## Mechanisms of QS inhibition—QQ

**Fig. 1:**
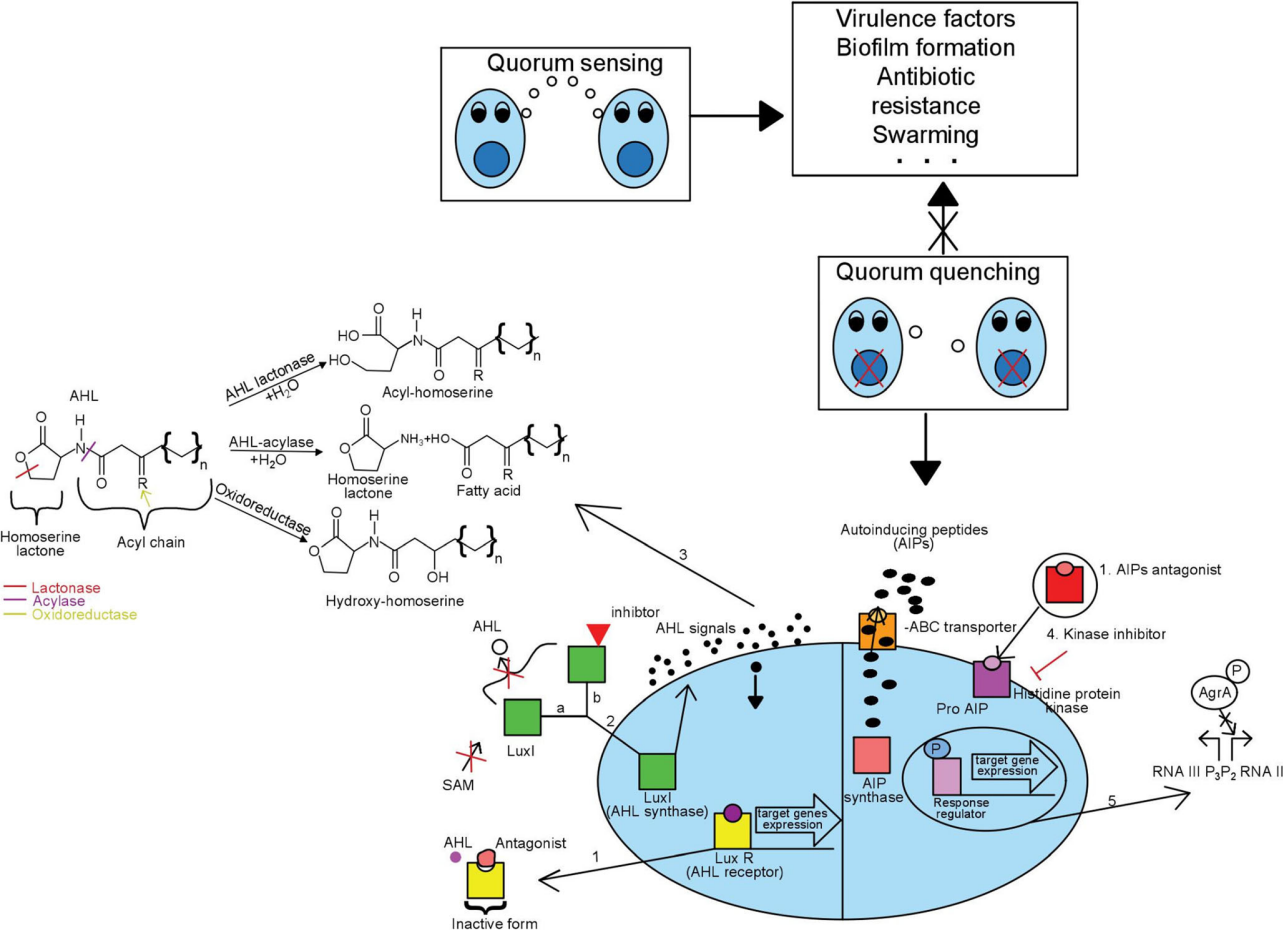
Mechanisms of blocking quorum sensing in Gram-negative and Gram-positive bacteria. Cell-cell communication in many bacteria is responsible for the production of various virulence factors. Disturbances in quorum sensing will inhibit the production of virulence factors. QS systems differ in Gram-negative and Gram-positive bacteria. In Gram-negative bacteria, the role of autoinductors is played by AHLs, synthesized by a LuxI type enzyme. These molecules penetrate the bacterial cell membrane, and after reaching the appropriate threshold concentration, the LuxR receptor protein is activated and transcription of target effector genes occurs. Signal molecules in Gram-positive bacteria are AIPs. They are synthesized in the form of pre-peptides, and after modification they are exported outside the cell via the ABC-ATP binding cassette transport system. After reaching the threshold concentration in the environment, the autoinducer molecules are bound by sensor proteins with kinase activity. Kinase is activated by phosphorylation. The phosphate group is transferred to the transcription regulator, which results in activation of the transcription of the target genes. Mechanisms interfering with QS cascades are marked with numbers on the diagram: 1—application of inductor antagonists; 2—inhibition of AHL molecule synthesis ((a)blocking SAM biosynthesis (b) inhibiting LuxI); 3—enzymatic degradation of AHL molecules (lactonase—hydrolyzes the HSL ring; acylase—hydrolyzes the amide bonds; oxidoreductase—reduces carbonyl or hydroxyl groups); 4—inhibition of histidine protein kinase activation by kinase inhibitor; 5—blocking of signal transduction cascades (inhibition of RNA III production by disturbing AgrA DNA binding)

There are several main mechanisms of QS inhibition (Fig. 1):

- Inhibition of signal molecule synthesis (e.g., blocking of Lux operon proteins) (Lade et al. 2014);
- Inactivation or enzymatic degradation of signal molecules (Lade et al. 2014; Rampioni et al. 2014; Delago et al. 2016);
- Competing with signal molecules–receptor analogues (Ni et al. 2009);
- Blocking the signal transduction cascades (e.g., by blocking AI-receptor complex formation (Rampioni et al. 2014).

The best known mechanism of quorum quenching is the enzymatic degradation of AHL molecules, which may be catalyzed by four distinct groups of enzymes: lactonases and acylases hydrolize the HSL ring and amide bond of AHL, respectively, while reductases and oxidases modify the activity of AHL, but do not degrade it (Rehman and Leiknes 2018). Another mechanism that interferes with communication between bacteria depends on the use of inductor antagonists. Such molecules can bind to the receptor by competing with inductors for the same binding site, or they bind the receptor non-competitively to block the inductor-mediated signal transmission into the cell (Bodede et al. 2018). Different QQ approach is the inhibition of the synthesis of signal molecules, such as AHL, by C8-HSL that impedes the enzymatic activity of LuxI (Hirakawa and Tomita 2013). Kinase inhibitors, e.g., closantel, RWJ-49815, and LY266500, result in inhibition of the QS signal molecules in Gram-positive bacteria (Brackman and Coenye 2015a). The last mechanism is blocking the signal transduction cascades. It has been shown that savrin, a small molecule inhibitor, interferes with AgrA (transcriptional regulator of the QS-involved *agr* operon), binds to DNA and, as a result, inhibits the production of RNAIII which, together with AgrA, is responsible for the production of many virulence factors (Sully et al. 2014).

## Prevention of virulence factor synthesis and biofilm production by quorum quenching

Production of virulence factors such as pyocyanin or pyoverdine in *P. aeruginosa* may be inhibited by different compounds. Such activity was proved for some quaternary ammonium salts, especially those with more than one hydrophilic group (Piecuch et al. 2016). As it was mentioned above virulence may be QS-dependent so using quorum quenchers may help in achieving a desired effect by the disruption of bacterial communication, especially when disinfectants and antibiotics are ineffective because of the growing bacterial resistance to such agents.

The quorum quenching phenomenon consists in the enzymatic degradation of signal molecules of the quorum sensing system in order to avoid their accumulation in the environment and inhibit the change in gene expression. These enzymes, due to inhibition of the production of autoinductors, are able to prevent the production of virulence factors, including biofilms produced by drug-resistant bacteria. The AHL-lactonase enzyme produced by *Bacillus cereus* VT96 directly controls the production of virulence factors such as exopolysaccharide production, biofilm formation, and pyocyanin production in *Pseudomonas aeruginosa* PAO1 (Rajesh and Rai 2016).

**Fig. 2:**
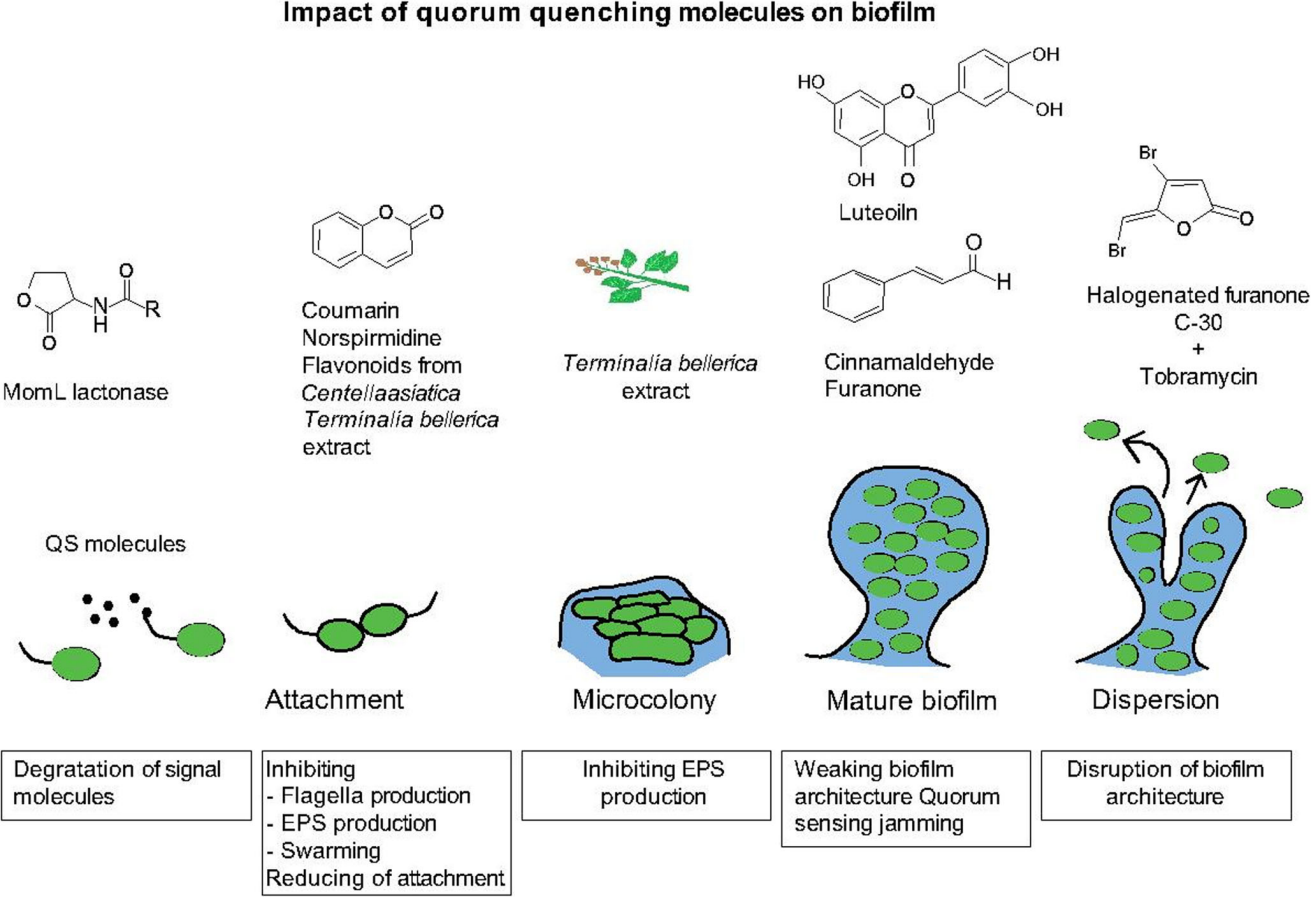
Impact of quorum quenching molecules on biofilm formation. QS affects various bacterial behaviors including biofilm formation, EPS production, and dispersion. Interference in communication between bacteria affects in the various biofilm stages. Degradation of signal molecules results in insufficient number of inductors to reach threshold concentration, thus inhibiting the whole QS process. Examples of QQ enzymes that degrade the signal molecules are lactonases and acylases. The use of QS inhibitors such as C8-HSL inhibits the production of virulence factors facilitating attachment. Quorum sensing inhibitors cause loosening of the biofilm structure by reducing the hydrophobicity of cell surface. The synergistic use of QSI with antibiotics was successfully applied to target mature biofilm

Some bacterial species such as *Pseudomonas aeruginosa*, *Klebsiella pneumoniae*, *Bacillus* spp., and *Agrobacterium tumefaciens* produce enzymes that are capable of degrading AHL molecules. MomL lactonase isolated from *Muricauda olearia* Th120 has the ability to degrade both long- and short-chain AHL equally, thus inhibiting virulence in many pathogenic bacteria (Wang et al. 2019a).In addition, the ability to produce enzymes that degrade AHL molecules and thereby interfere with QS has also been found in plants and fungi such as *Pachyrhizus erosus*, *Lotus corniculatus*, *and Hordeum vulgare*. Medicinal plants produce a wide spectrum of secondary metabolites such as flavonoids, phenols, phenolic acids, saponins, coumarins, tannins, quinones, terpenoids, alkaloids, and polyacetylenes, against the QS system. The differentiation in inhibition of QQ molecules depends on the structure and chemical composition of the compound. For example halogenated furanones, produced by the *Delisea pulchra* marine macroalga, were the first anti-QS compound identified which activity was based on competitive binding to LuxR-type proteins. This mechanism of action leads to increased proteolytic degradation of the LuxR type proteins (Asfour 2018). It has also been proved that natural compounds—embeline and piperine—inhibit the production of biofilm by *Streptococcus mutans*, by inhibiting the activity of receptors and molecules involved in the QS pathway (Dwivedi and Singh 2016). Extract from the *Terminalia bellerica* plant effectively inhibited the production of pyocyanin and EPS in *P. aeruginosa*, whose production is under the control of the QS communication system (Sanker Ganesh and Ravishankar Rai 2017). Husain and his team have demonstrated the inhibitory effects of PMO (peppermint oil) and menthol on QS by inhibiting the violacein produced by *C. violaceum*, which is under the control of AHL. These compounds also resulted in inhibition of pyocyanin, elastase, protease, EPS production and biofilm production by *P. aeruginosa* (Husain et al. 2015). Flavonoids are compounds found in plants that can interfere with communication between microorganisms and have an anti-biofilm effect. Isolated flavonoids from *Centella asiatica* showed inhibitory effects on swarming and twitching motility, and production of pyocyanin and biofilm in *P. aeruginosa* PAO1 (Vasavi et al. 2016). Other example is coumarin, which is commonly found in plants, reducing the production of virulence factors such as the production of pyocyanin, protease, and biofilm by interfering with QS in *Pseudomonas aeruginosa* (Zhang et al. 2018). Anti-biofilm activity was also observed for *Ananas comosus* extract (pineapple) or *Musa paradiciaca* (banana) water extracts which prevented the synthesis of *P. aeruginosa* virulence factors such as proteases, elastases, and pyocyanin what resulted in decreased biofilm production (Musthafa et al. 2010). Similar activity was also found for grapefruit extract containing furocoumarins that disrupt AI 1– and AI 2–based QS in *P. aeruginosa* and *S*. Typhimurium (Girennavar et al. 2008). Curcumin-rich turmeric (*Curcuma longa*) prevents biofilm formation at early stages (Rudrappa and Bais 2008). Anti-biofilm QQ-dependent activity of secondary plant metabolites (quercetin, apigenin, naringenin, and kaempferol) was also found against *E. coli* O157:H7 (Kalia 2013). Vikram et al. (2010) proved anti-biofilm effect in *Yersinia enterocolitica* after exposition to orange extract rich in flavons (hesperidin, neohesperidin, and naringenin) which lowers an amount of produced AHLs (Vikram et al. 2010). The impact of QQ molecules on biofilm production is illustrated below (Fig. 2).

Synthetic QQ molecules such as cinnamyl alcohol, allyl cinnamate, and methyltrans-cinnamate, which are derivatives of cinnamic acid inhibited the production of the important virulence factor, violacein, by *C. violaceum* (Chen et al. 2019; Wang et al. 2019b). Norspirmidine, a type of polyamine, has reduced the expression of lasI, lasR, rhlI, rhlR, and mvfR genes that are involved in the QS system in *P. aeruginosa.* This led to reduced attachment to the surface. A smaller number of cells attached to the surface resulted in limited biofilm production and easier eradication (Qu et al. 2016). Biofilm formation can be also prevented by applying the analogues of AHL molecules that inhibit QS communication. The effectiveness of such strategy has been demonstrated against *Acinetobacter baumanii* (Chow et al. 2014). QQ molecules can also reduce the production of exopolysaccharide (EPS) which is an important component of bacterial biofilm, crucial to maintain the spatial structure of the consortium. Decrease in its production may results in the formation of impaired biofilms (Husain et al. 2015). Naringenin, through downregulation of the expression of the lasI and rhlI genes and inhibition of LuxR transcription factors, leads to reduced production of AHL molecules. LasI and rhlI mutants that are deficient in the synthesis of AHL molecules have a lower ability to express a wide spectrum of QS genes (Vandeputte et al. 2011). Paraoxonases present in several human cell lines and mammalian serum are also extremely important, as they also have the ability to degrade AHL molecules (Brackman and Coenye 2015b; Chang et al. 2019).

## Methods used to analyze—QQ and QS

Many researchers focus on discovering or synthesizing effective QS inhibitors and try to investigate their properties. Many QQ molecules have been characterized so far, and it should be emphasize that finding a molecule that will target all the abovementioned QS mechanisms is quite unlikely. However, there are some criteria gathered by Kalia (2013) that may help researchers in searching for effective QS inhibitors. First is low molecular weight and chemically stable structure. Moreover, the ability to decrease the expression of genes that are under QS regulation is very important. Furthermore, QQ should be highly specific to recognize and inhibit particular autoinductors. The most important feature in the context of medical application is the lack of host cell cytotoxicity and metabolic neutrality. Fulfillment of as many of these conditions as possible may help avoid bacterial resistance to the QQ-based treatment and host adverse effects (Kalia 2013).

Basic methods of searching for bacteria with QQ activity can be divided into three groups: residual AHL measurement, plate inhibition assay, and minimum medium assay (Tang et al. 2013). The techniques, which are now commonly used for quantitative and qualitative analysis of QQ and QS molecules involve colorimetry, bioluminescence, chemiluminescence, fluorescence, chromatography-mass spectroscopy, and electrochemistry (Van der Meer and Belkin 2010).

### Measurement of AI production

Quantitative and qualitative AI measurement may be performed by variety of different techniques that may be classified as direct or indirect (biosensor is needed). Most of them are based on detection of signaling molecules that possess functional groups that react with specific chemicals giving color reaction which may be quantified (colorimetry) or have bioluminescence ability. Other used methods are HPLC, GC-MS, or LC-MS methods (Yang et al. 2006). Liquid chromatography coupled with tandem mass spectrometry (LC-MS/MS) may detect different AHLs at the same time what is an advantage of this method (Cataldi et al. 2013). This method has also limitations: only molecules with the concentration of above 0.6 nM could be detected so such method cannot be used for C10-HSL detection (Patel et al. 2016). Tandem mass spectrometry was also used to detect farnesol and tyrosol, QS molecules produced by *Candida albicans* (Greguš et al. 2010). There is also an opportunity to detect production of other molecules, e.g., peptides. More than 200 signaling peptides engaged in QS process are known as well as their structural analogues. An important peptide is competence stimulating peptide (CSP) involved in the QS of *S. mutans*. The CSP is a peptide pheromone where the competence regulon engaged in the regulation of biofilm formation, stress response, and bacteriocin production is centered. To characterize the structure of peptides LC-MS method may be used (Wynendaele et al. 2013; Verbeke et al. 2017; Bikash et al. 2018; Debunne et al. 2018).

Among others, methods based on using so-called whole-cell bacterial biosensors are reported for AHL detection. They may be defined as genetically modified organisms such as *Vibrio fischeri*, *Pseudomonas aeruginosa*, or *Agrobacterium tumefaciens* bacteria that possess a reporter coupled with a biological recognition agent and have the ability to use bacterial pathways and proteins to detect bacterial QS molecules. The reporter protein may be detected optically or electrochemically; for example, the measurement of an amount of the protein luciferase encoded by *lux* gene can be quantified via bioluminescence. QS biosensors serve as fast and reliable tools for detecting specific or various types of autoinductors (Wynendaele et al. 2013; O’Connor et al. 2015; Rai et al. 2015). Examples of methods based on biosensors are listed below (Table 1).To detect AHLs, mutants of bacteria strains that naturally produce bioluminescence in response to AHL molecules may also be used. The most commonly performed bioluminescence assay is *Vibrio harveyi* BB170 test. This strain has a mutation in the genome (*luxN*::tn5Kan); hence, it is unable to produce AHLs and AI-2, because *luxN* gene encoding LuxN protein was deleted. This strain is also impaired in AHL detection; thus, bioluminescence may only be observed when exogenous AI-2 is present in bacterial environment. Bioluminescence intensity is correlated with an amount of tested autoinductors (O’Connor et al. 2015). Genetic modifications could also be used to construct bacterial strains with specific QQ activity. That may be obtained by inserting genes (e.g., *aiiO* gene encodes AHL degrading enzyme—AHL lactonase) into competent cells’ genomes as was described by Oh et al. (2017). The strains modified in that way gain QQ activity.

**Table 1:** Quorum sensing peptide biosensor detection systems (Verbeke et al. 2017)

Quorum sensing molecule	Amino acid sequence	Species	Function	LOD (limit of detection)
CSP	DSRIRMGFDFSKLFGK	*S. anginosus*, *S. thermophilus*, *S. constellatus*	Biofilm formation assay	0.2 nM
CSP	DRRDPRGIIGIGKKLFG	*S. milleri*	Transformation assay	105 nM
CSP	EMRISRIILDFLFLRKK	*S. pneumoniae*	Transformation assay	46 nM
CSP	EMRLSKFFRDFILQRKK	*S. pneumoniae*	Transformation assay	45 nM
CSP	SQKGVYASQRSFVPSWFRKIFRN	*S. gordonii*	Transformation assay	72 nM
21-CSP	SGSLSTFFRLFNRSFTQALGK	*S. mutans*	Β-galactosidase assay, bacteriocin production, transformation assay	Not specified
EDF	NNWNN	*E. coli*	Colony forming unit assay	150 nM
EntF	AGTKPQGKPASNLVECVFSLFKKCN	*E. faecium*	Bacteriocin induction	10 aM
cAD1	LFSLVLAG	*E. faecalis*	Microtiter dilution method	50 pM
cAM373	AIFILAS	*E. faecalis*	Microtiter dilution method	50 pM
Gelatinase biosynthesis-activating pheromone	QNSPNIFGQWM	*E. faecalis*	Bioluminescence	Not specified
cCF10	LVTLVFV	*E. faecalis*	Clumping induction assay	25 pM
cPD1	FLVMFLSG	*E. faecalis*	Clumping induction assay	40 pM
cOB1	VAVLVLGA	*E. faecalis*	Clumping induction assay	Not specified
iAD1	LFVVTLVG	*E. faecalis*	Microtiter dilution method	Not specified
iPD1	ALILTLVS	*E. faecalis*	Microtiter dilution method	Not specified
iCF10	AITLIFI	*E. faecalis*	Inhibition of induced self-clumping	10 nM
iAM373	SIFTLVA	*E. faecalis*	Clumping induction assay	200 pM

Techniques based on enzymatic activity measurement may also be applied for AHL detection. The most common QQ-inducing enzymes are AHL-acylase, AHL-lactonase, oxidoreductase, and paraoxonase. They may be active only against specific QS molecules or have broad spectrum of activity (Chen et al. 2013). An important step in QQ research is a preliminary mapping of QQ enzymes by using the BLASTP database, followed by the enzyme purification and characterization (Tang and Zhang 2018). The ability of QQ enzymes to reduce virulence of bacterial strains may also be tested by modifying bacterial genome by introducing specific genes (e.g., AHL-lactonase gene *aiiA*). One such test was performed using *Burkholderia glumae*, a common rice QS-dependent pathogen. After transformation the pathogenicity was significantly reduced (Park et al. 2010; Chen et al. 2013). To detect residual AHLs the A136 liquid X-gal assay (using *Agrobacterium tumefaciens* A136 strain) may also be applied. The method described by Zhu et al. (1998) with the modifications by Tang et al. (2013) is based on measurement of β-galactosidase activity. This test, because of the usage of liquid medium X-Gal A136 with β-galactosidase, enables not only detection of AHLs but also determination of their activity level by colorimetric measurement (Tang et al. 2013).

### Minimum medium assay

Minimum medium assay is a quick technique used for the isolation of bacteria with the QQ ability from natural sources. This method is based on usage of AHLs by bacteria as an only source of biogenic elements and energy (Tang et al. 2013). According to the protocol given by Chan et al. (2009), minimum medium supplemented with AHLs, as the source of nitrogen and carbon, may be used to isolate bacteria with the ability to degrade such molecules because the growth of bacteria unable to resolve them cannot survive in such conditions. Unfortunately, despite effects presented in previous publications (Christiaen et al. 2011) this method has some limitations. Researchers found that the most bacterial species, despite having AHL-degrading activity, are unable to grow in medium poor in nutrients and other crucial components so it cannot be considered universal method (Chan et al. 2009; Uroz et al. 2009). Minimum medium assay should be used as an initial screening method and be followed by other research because it does not allow to determine what QQ molecules these bacteria use and how.

### Plate diffusion assay

Another method used for an identification of QQ-producing bacteria is plate diffusion assay. The principle of this method is as follows: QS-interfering molecules produced by tested bacterial stain can penetrate through the solid medium, and to detect them a biosensor strain is needed. The most common biosensor strains are *Agrobacterium tumefaciens* A136 and *Chromobacterium violaceum* O26 (McLean et al. 2004). According to the protocol given by McLean et al. (2004) this method may be performed as follows: tested bacterial strain is streaked onto the central part of the agar plate and, after incubation, overlaid with semi-solid medium containing biosensor strain. QS inhibition can be detected when zones of pigment production inhibition emerge in the vicinity of tested bacterial spot (McLean et al. 2004; Tang et al. 2013). Unfortunately this method has limitations: it is time- and labor-consuming, and the only parameter that is measured is inhibition zones what can be imprecise and a strong QQ activity is needed to observe them (Uroz et al. 2009; Liu et al. 2010; Tang et al. 2013; Lee et al. 2016).

## Applications of QQ

The biotechnological applications of quorum sensing inhibitors (quorum quenchers) have been frequently reported in recent publications, and the usage of different quorum quenchers has been described so far (Kalia et al. 2019). They may constitute products of bacteria (e.g., norspermidine), plants (secondary metabolites, e.g., catechins from green tea leaves *Camellia sinensis*), and animals (enzymes such as acylases, lactonases, and oxidoreductaes isolated from *Mus musculus* or *Danio rerio* (Kalia 2013; Nesse et al. 2015; Yin et al. 2015; Qu et al. 2016). QQ molecules may be applied in variety of fields, especially in medicine and biotechnology.

### Medical application

Spreading of multi-drug-resistant bacteria (*Klebsiella pneumoniae* New Delhi metallo-beta-lactamase 1 (NDM-1) or *Staphylococcus aureus* MRSA) in hospitals is a crucial problem that has to be faced immediately (Walsh and Toleman 2012).Some microorganisms are etiological factors of serious and hard to combat diseases. Such strains produce numerous virulence factors and form biofilms that are difficult to eradicate. Searching for chemicals with the ability to prevent microbial adhesion (biofilm prevention) and combat pre-formed biofilm is crucial in variety of fields. One of them is health care, where diseases based on biofilm formation (oral cavities, cystic fibrosis, and others) are a serious problem. An effective QS inhibition may be very helpful for patients suffering from such condition (Yada et al. 2015). Promising results were published by Utari et al. (2018) who studied an activity of PvdQ acylase on AHL molecules of *Pseudomonas aeruginosa* (a common pulmonary pathogen) in a mouse model which resulted in the decrease of infection (Utari et al. 2018). *P. aeruginosa* PAO1 virulence was also studied by Vandeputte et al. (2010) who proved that specific flavanoids are able to decrease signal perception what results in lower virulence and inhibition of biofilm formation (Vandeputte et al. 2010). AHL molecules may also be modified by formation of synthetic structural analogues with modified lactone rings and functional groups. Structural similarity enables binding with QS receptor but does not lead to its activation so transcription factors are not expressed. 4-Nitro-pyridine-N-oxide (4-NPO), a QS inhibitor, influences the virulence of *P. aeruginosa* by downregulation of virulence-involved genes (*lasA*, *lasB*, *chiC*, and *rhlAB*) and reduces a tobramycin tolerance in the biofilm (Rasmussen et al. 2005). *Staphylococcus aureus* is a common pathogen with the ability to form biofilms; thus, the activity of QQ molecules may be used to inhibit its virulence (Ziemichód and Skotarczak 2017). Lactonase isolated from *Geobacillus kaustophilus* HTA426 is reported to degrade lactone ring in the AHL’s structure which affects *Acinetobacter baumannii* by impeding the biofilm production (Chow et al. 2014). A promising idea may be the combination of quorum quenchers and common antibiotics. QQ molecules do not kill bacteria themselves but influence their virulence factors in different ways what weaken the pathogens (Rasmussen et al. 2005).

### Biotechnological application

Quorum sensing inhibitors are also widely reported in agriculture. Epiphytic bacteria (e.g., *Pseudomonas*) reduce plant infections by influencing QS of pathogens (Dulla and Lindow 2009). Compounds isolated from *Streptomyces xanthocidicus* are reported to protect potatoes from soft rot by an ability to compete for the binding site of AHL in *Erwinia carotovora* (Kang et al. 2016). Another example of QQ application is aquaculture where AHL lactonase isolated from *Bacillus* sp. is reported to inhibit *Danio rerio* infections or cinnamaldehyde isolated from the cinnamon bark protects prawns from *Vibrio harveyi* infection (Chu et al. 2014; Pande et al. 2013). Biofilm formation may be also problematic in water treatments where bacteria adhere to membrane filters disrupting their work. Using selected bacterial strains with the ability to degrade QS molecules is crucial. Yavuztürk Gül and Koyuncu (2017) reported QQ activity of *Bacillus* sp. (Yavuztürk Gül and Koyuncu 2017). Yu et al. (2016) investigated biostimulation process (with gamma-caprolactone (GCL) as stimulant) as a promising strategy for prevention of biofilm formation in membrane bioreactors (Yu et al. 2016). Transmembrane pressure is an important factor that indicates the effectiveness of the filtration process. Pervez et al. (2018) revealed that the usage of immobilized *Enterobacter cloacae* and *Rhodococcus* sp. BH4 can prolong the filtration time from 12 to 27 and 17 days respectively because of the disruption of EPS production on the membrane surface (Pervez et al. 2018). Zhang et al. (2019) examined a photolytic QQ—the impact of UV on QS molecules—and suggested that this may be applied in water treatments because of the satisfying effectiveness in QQ. Biofilm formation is also a problem in metalworking systems. Özcan et al. (2019) proved that using patulin and furanone C-30 on such utensils resulted in 63% and 76% *P. aeruginosa* PAO1 biofilm reduction, respectively.

## Alternative QQ strategies

Nanotechnology-based clinical strategies are gaining a lot of interest. Nanomolecules as well as nano- and microcomposites, e.g., Ag- or ZnO-based compounds, were reported to be effective quorum quenchers due to their ability to inhibit microcolony formation that resulted in the decreased biofilm production and alteration of its structure. Silver-based nanoparticles have strong antibacterial activity against *E. coli* planktonic forms as well as mature biofilm. The activity of nanoparticles may be linked to the degradation of receptor proteins or inhibition of AI molecule synthesis that results in decrease production of virulence factors, such as elastase, pyocyanin, and biofilm components (Radzig et al. 2013; Garcia-Lara et al. 2015).

Apart from their usefulness in antimicrobial therapies, QQ may be also applied in cancer treatment. Li et al. (2004) proved that 12-C AHLs inhibit proliferation and promote apoptosis of breast cancer cells. Unfortunately some of the tested QQ (e.g., halogenated furanones) are cytotoxic to normal cells (Li et al. 2004; Bjarnsholt and Givskov 2007).
